# Identification of the immune-associated characteristics and predictive biomarkers of keratoconus based on single-cell RNA-sequencing and bulk RNA-sequencing

**DOI:** 10.3389/fimmu.2023.1220646

**Published:** 2023-10-27

**Authors:** Xiaoguang Niu, Man Xu, Jian Zhu, Shaowei Zhang, Yanning Yang

**Affiliations:** ^1^Aier Eye Hospital of Wuhan University, Wuhan, China; ^2^Department of Ophthalmology, Renmin Hospital of Wuhan University, Wuhan, China; ^3^Hanyang Aier Eye Hospital, Wuhan, China

**Keywords:** Keratoconus, single-cell RNA sequencing, immune-associated genes, corneal stromal cell, diagnostic biomarkers

## Abstract

**Background:**

Whether keratoconus (KC) is an inflammatory disease is currently debated. Hence, we aimed to investigate the immune-related features of KC based on single-cell RNA sequencing (scRNA-seq) and bulk RNA sequencing (bulk RNA-seq) data.

**Methods:**

scRNA-seq data were obtained from the Genome Sequence Archive (GSA), bulk RNA-seq data were obtained from the Gene Expression Omnibus (GEO), and immune-associated genes(IAGs) were obtained from the ImmPort database. Cell clusters of KC were annotated, and different cell clusters were then selected. The IAG score of each cell was calculated using the AUCell package. Three bulk RNA-seq datasets were merged and used to identify the differentially expressed genes (DEGs), biological functions, and immune characteristics. Weighted gene coexpression network analysis (WGCNA) was used to select the IAG score-related hub genes. Based on scRNA-seq and bulk RNA-seq analyses, three machine learning algorithms, including random forest (RF), support vector machine (SVM), and least absolute shrinkage and selection operator (LASSO) regression analysis, were used to identify potential prognostic markers for KC. A predictive nomogram was developed based on prognostic markers.

**Results:**

Six cell clusters were identified in KC, and decreased corneal stromal cell-5 (CSC-5) and increased CSC-6 were found in KC. CSC and immune cell clusters had the highest IAG scores. The bulk RNA-seq analysis identified 1362 DEGs (553 upregulated and 809 downregulated) in KC. We found different immune cell populations and differentially expressed cytokines in KC. More than three key IAG score-related modules and 367 genes were identified. By integrating the scRNA-seq and bulk RNA-seq analyses, 250 IAGs were selected and then incorporated into three machine learning models, and 10 IAGs (CEP112, FYN, IFITM1, IGFBP5, LPIN2, MAP1B, RNASE1, RUNX3, SMIM10, and SRGN) were identified as potential prognostic genes that were significantly associated with cytokine and matrix metalloproteinase(MMP)1-14 expression. Finally, a predictive nomogram was constructed and validated.

**Conclusion:**

Taken together, our results identified CSCs and immune cell clusters that may play a key role during KC progression by regulating immunological features and maintaining cell stability.

## 1 Introduction

Keratoconus (KC) is a disease characterized by bilateral progressive corneal ectasia, which is caused by established biomechanical instability. This disease is mainly identified by thinning of the corneal stroma and asymmetric conical protrusions (1). KC can cause myopia, irregular astigmatism, and significant visual function impairment. The prevalence of KC varies considerably among different populations. One of the lowest incidence rates (0.0068%) has been reported in North Macedonia (2), and the highest prevalence rate (4.79%) is found among paediatric patients in Saudi Arabia (3). According to a meta-analysis, the frequency of KC in the general population is estimated to equal 1.3/1000 (4). Moreover, the prevalence of KC among young adults is approximately 1 in 84 to 1 in 375 (5, 6). With the development of corneal topography and optical coherence tomography (OCT), the diagnosis of KC has significantly improved, leading to earlier and more accurate detection. Consequently, the incidence of KC may exceed previously established levels.

The exact cause of KC is unclear, and it is generally believed to be more related to genetic and environmental factors (7). Genetic disorders, such as Down syndrome, as well as environmental factors, including eye rubbing, atopy, and ultraviolet exposure (8, 9), can cause abnormal remodelling of the extracellular matrix in corneal tissue (10). KC has been defined as a noninflammatory eye disease (11), mainly due to a lack of inflammation-related manifestations in the corneas of KC patients. However, an increasing number of recent studies have confirmed that immunity and inflammation play an important role in the pathogenesis of KC (12). Differential expression of immune cells and inflammatory factors has been observed in the ocular surface and circulatory system of patients with KC (13), and patients with immune abnormalities such as atopic allergies also exhibit a significantly increased risk of developing KC (14). Multiple inflammatory factors in the cornea and tears of patients with KC are significantly increased, and some inflammatory factors are unique to KC. IL-10, IL-6, MMP-9, MMP-1, HGF, VEGFA, MMP-3, MMP-2, TGFB1, IL-4, IL-2, and IFNG are key inflammatory factors in KC, and the IL-17 signalling pathway may play an important role in the pathogenesis and development of KC (15). Despite evidence of inflammation accumulated from some studies, the histological and clinical features of inflammation in KC, such as cell infiltration and neovascularization, remain strikingly lacking (16).

With increasing next-generation sequencing (NGS) technology, several molecular biomarkers and pathways that are involved in the stimulation of inflammation have been found, including the monocyte-to-HDL-cholesterol ratio (MHR), neutrophil-to-lymphocyte ratio (17), and PTEN-putative kinase-1 (PINK1) (18). However, the immune characteristics of KC are poorly understood. Immunity-associated genes (IAGs) play essential roles in immune infiltration; the expression characteristics of IAGs and possible regulatory mechanisms of immune infiltration in KC remain unclear. We identified IAG score-related gene modules and possible regulatory mechanisms in KC through bioinformatic analysis combining single-cell RNA (scRNA) and bulk sequencing data. Moreover, we also compared immune cell populations and cytokines as well as MMPs between KC patients and healthy controls.

## 2 Materials and methods

### 2.1 Data sources and processing

Bulk RNA-seq data of KC patients were obtained from the GSE151631, GSE112155, and GSE77938 datasets of the Gene Expression Omnibus (GEO, https://www.ncbi.nlm.nih.gov/). The GSE151631 dataset included 19 cornea specimens from KC patients and 7 cornea specimens from healthy controls (19), the GSE112155 dataset included 10 cornea specimens from KC patients and 10 cornea specimens from healthy controls (20), and the GSE77938 dataset included 25 cornea specimens from KC patients and 25 cornea specimens from healthy controls (21); these three datasets were generated using the GPL16791, GPL18573, and GPL18460 platforms, respectively. In the current study, the transcriptome data from the abovementioned three datasets were merged by eliminating batch effects using the combat function of the “sva” package. Principal component analysis (PCA) was performed to evaluate the combat performance. In addition, a total of 1242 immune-associated genes (IAGs) were obtained from the Immunology Database and Analysis Portal (ImmPort, https://www.immport.org/home) (22).

### 2.2 ScRNA-seq data processing

Single-cell RNA sequencing data of cornea specimens from KC patients and healthy controls in the HRA000728 dataset were downloaded from the Genome Sequence Archive (GSA, https://ngdc.cncb.ac.cn/gsa/) (23). The expression profiles were processed using the “Scanpy” package of Python. The low-quality cells were filtered out with the following criteria: cells with a total number of molecules per cell less than 5, a unique gene number per cell lower than 500 and greater than 5000, and a percentage of mitochondrial genes higher than 5%. The filtered cells were selected for subsequent analyses. The expression profiles were normalized using normalized_total, loglp, and scale functions, and 3000 highly variable genes (HVGs) with highly variable gene functions were then identified for each sample. Afterwards, the data were processed by homogenization using the “harmony” package, principal component analysis (PCA), and uniform manifold approximation and projection (UMAP) analysis. The optimal number of principal components (PCs) was determined with ElbowPlot, and the positional relationship between each cluster was determined by setting the resolution value to 1 and visualized using UMAP analysis. The rank_genes_groups function was applied to identify the differentially expressed genes (DEGs) for each cluster, and marker genes of each cluster were identified with the criterion P value < 0.05. Each cluster was annotated and manually checked by referencing the CellMarker2 database (http://xteam.xbio.top/CellMarker/) and a previous article (23) using Leiden clustering.

### 2.3 IAG score of each cell in KC

The DEGs between KC patients and healthy controls were identified using the scanpy.tl.rank_genes_groups function with the following thresholds: |log2 (fold change, FC)| > 1 and adjusted P < 0.01. The differentially expressed IAGs in KC were screened out from the abovementioned DEGs based on the ImmPort database (https://www.immport.org/home). The AUCell package was used to calculate the IAG score of each KC cell.

### 2.4 Functional enrichment analysis

The ClusterProfiler package was used to perform the functional enrichment analysis, which includes Gene Ontology (GO) annotation and Kyoto Encyclopedia of Genes and Genomes (KEGG) pathway enrichment. GO annotation comprises biological processes (BPs), molecular functions (MFs), and cellular components (CCs). The p value was adjusted using the Benjamini-Hochberg method or corrected using the false discovery rate (FDR). The top 10 pathways were identified through their p value-based ranking.

### 2.5 Identification of DEGs between KC and control based on the GEO database

After batch effect removal using the combat function of the sva package, mRNA profiles from the GSE151631, GSE112155, and GSE77938 datasets were used to screen the DEGs between KC patients and healthy controls. The limma package was then used to identify DEGs based on the following criteria: |log2 FC| > 0.585 and FDR < 0.05.

### 2.6 Immune cell infiltration analysis

The marker genes of different immune cell types were obtained from a previous article (24). The abundance of immune cells was then evaluated using the xCell package in R. The differences in the immune cell proportions between KC patients and healthy controls were determined by the Kruskal-Wallis test. Moreover, we investigated the differences in 24 proinflammatory cytokines (IL-1B, IL-4, IL-5, IL-6, IL-9, IL-13, IL-18, LIF, IL-17A, TNF, IFNA1, IFNA2, IFNG, CRP, EPO, TGFB1, PDGFB, VCAM1, SELL, GZMB, PRF1, FASLG, TLR2, and TLR4) and MMPs (MMP1-14) between KC patients and healthy controls using the Kruskal-Wallis test.

### 2.7 Construction of a weighted gene coexpression network analysis

According to the above-described immune cell infiltration analysis, the IAG score of each sample was calculated. The WGCNA package was used to develop a coexpression network targeting DEGs. First, a weighted adjacency matrix was used to investigate the correlation between genes with a soft thresholding parameter. The adjacency was then converted into a topological overlap matrix (TOM) to measure the network connectivity of genes. The clustering tree structure of the TOM matrix was constructed using the agglomerative hierarchical clustering method. Different branches of the clustering tree represent different gene modules, and different colours represent different modules. Based on the IAG score of each sample and the weighted correlation coefficients of the top 25% of genes with median absolute deviation, genes with similar patterns were clustered into one module. Genes with module membership (MM) > 0.6 and gene significance (GS) > 0.3 were selected as immune-associated hub genes.

### 2.8 Identification of biomarkers for KC

The overlap between DEGs in the significant cell clusters with IAG scores identified from the scRNA-seq analysis and DEGs in the key modules identified by WGCNA was used to screen the hub IAGs in KC. Afterwards, three machine learning algorithms, namely, random forest (RF), support vector machine (SVM), and least absolute shrinkage and selection operator (LASSO) regression analysis, were used to select the biomarkers using the caret package. Biomarkers were then selected by overlapping the characteristic genes identified using the three abovementioned machine learning algorithms. The area under the receiver operating characteristic (ROC) curve (AUC) was used to evaluate the diagnostic efficacy of the biomarkers.

### 2.9 Construction of a nomogram

Diagnostic genes were incorporated to develop a nomogram using the regplot package. The calibration curve was drawn using the rms package to evaluate the accuracy of the nomogram. The clinical impact curve and decision curve were drawn using the dcurves package to evaluate the clinical usefulness of the nomogram.

## 3 Results

### 3.1 scRNA-seq data processing

**Figure 1:**
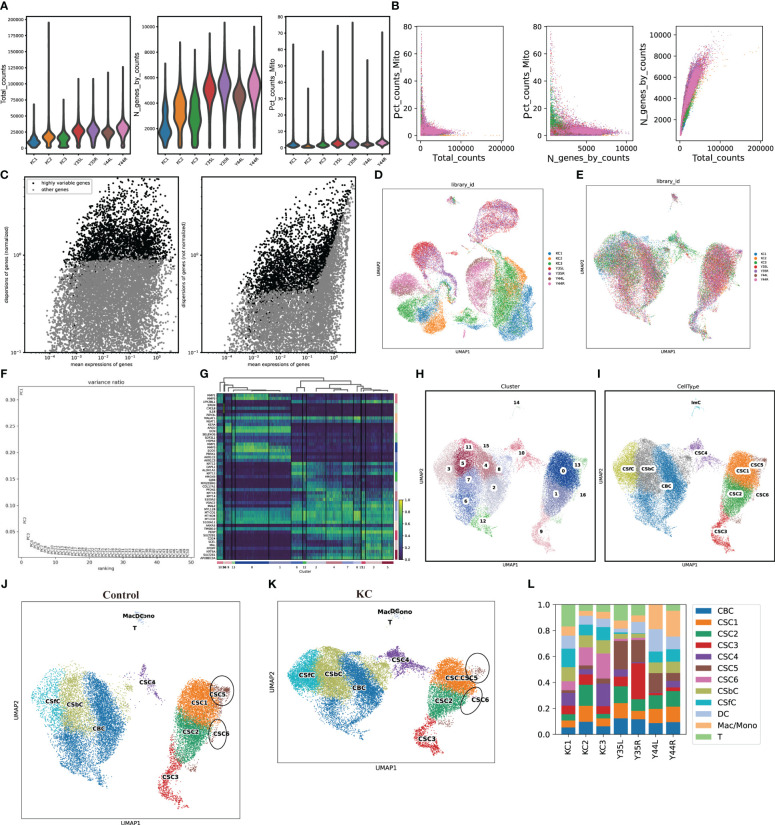
scRNA-seq data processing. **(A)** The violent plots of the genes (features), counts, and mitochondrial gene percentage of each sample. **(B)** Scatter plots of the correlation of mitochondrial genes with total counts, mitochondrial genes with gene counts, as well as gene counts with total counts of each sample, respectively. **(C)** Scatter plots of the top 3000 HVGs. **(D, E)** UMAP of a total of 50,283 cells and 43,256 cells before and after removing the batch effects. **(F)** Selection of 50 PCs. **(G)** Heatmap of the top three DEGs of each clearer. **(H, I)** UMAP of the distributed sixteen clusters and annotated with known cell lineages. **(J, K)** UMAP of the annotated cell clusters in health control and KC. **(L)** The distributions of cell clusters in each sample.

The scRNA-seq data were obtained from the GSA database, which included a total of 50,283 cells, including 27,572 cells from KC patients and 22,711 cells from healthy controls. After filtering, a total of 43,256 cells, which included 23,367 cells from KC patients and 19,889 cells from healthy controls, were used for subsequent analysis (**Figures 1A, B**). The top 3000 highly variable genes (HVGs) were identified (**Figure 1C**). The data were then successively processed by homogenization (**Figures 1C, D**). The top 50 PCs were identified and used for subsequent PCA (**Figure 1E**). Sixteen clusters were grouped using 50 PCs (**Figure 1F**), and the top three DEGs of each cluster are illustrated (**Figure 1G**). The sixteen clusters were annotated as known cell lineages by recognizing marker genes according to a previous article (23), and these lineages included corneal stromal cells (CSCs, 1-6), corneal epithelial cells (CECs, comprising corneal suprabasal cells (CSbCs), corneal basal cells (CBCs), and corneal superficial cells (CSFCs)), and immune cells (ImCs, comprising dendritic cells (DCs), T cells (Ts), and macrophages/monocytes (Macs/Monos)) (**Figures 1H, I**). The comparison of the cell clusters between KC patients and healthy controls revealed a decreased percentage of the CSC5 cluster and an increased percentage of the CSC6 cluster in KC patients compared with healthy controls (**Figures 1J–L**).

### 3.2 Transcriptional alterations in KC

**Figure 2:**
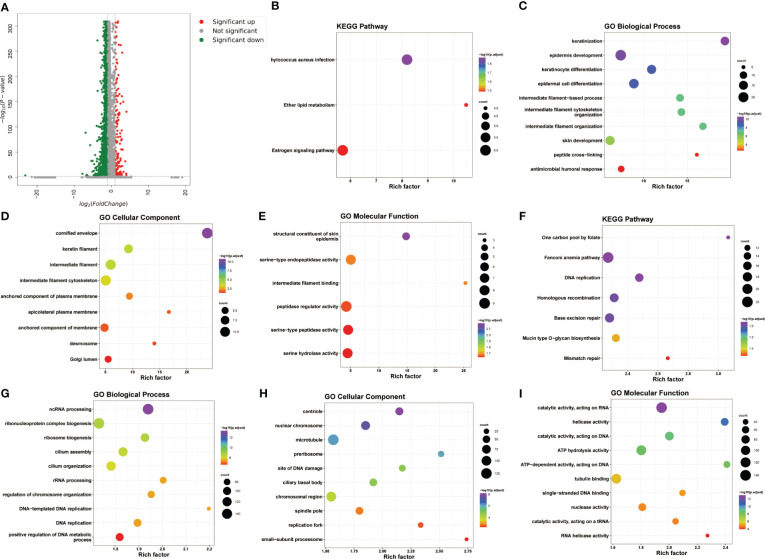
Transcriptional alterations in KC. **(A)** Volcano plot of DEGs between KC and health control based on the scRNA-seq data. **(B–E)** The bubble plots of KEGG pathways and GO annotation (BP, CC, and MF) of upregulated DEGs. **(F–I)** The bubble plots of KEGG pathways and GO annotation (BP, CC, and MF) of downregulated DEGs.

We next explored the transcriptional alterations in KC, and a total of 5608 DEGs (206 upregulated and 5402 downregulated) between KC patients and healthy controls were screened based on the criteria |log2FC| > 1 and adjusted P < 0.01 (**Figure 2A**). The upregulated DEGs were mainly enriched in the oestrogen signalling pathway, ether lipid metabolism, and Staphylococcus aureus infection (**Figure 2B**) and were mainly associated with the antimicrobial humoral response, peptide cross-linking, epidermal cell differentiation and development, epidermis development, Golgi lumen, desmosome, serine hydrolase activity, serine-type peptidase activity, and peptidase regulator activity, among other terms (**Figures 2C–E**). The downregulated DEGs were associated with mismatch repair, mucin-type O-glycan biosynthesis, base excision repair, etc. (**Figure 2F**). These downregulated DEGs were found to be involved in some biological processes, such as positive regulation of DNA metabolic process, DNA replication, and DNA-templated DNA replication (**Figure 2G**), and were associated with the small-subunit processome, RNA helicase activity, catalytic activity, acting on a tRNA, and nuclease activity (**Figures 2H, I**).

### 3.3 Identification of IAG score-specific cell types in KC

**Figure 3:**
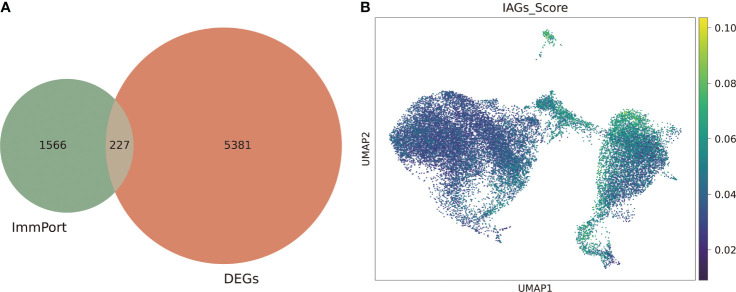
Identification of the IAG score-specific cell types in KC. **(A)** Venn plot of differentially expressed IAGs based on DEGs from scRNA-seq and ImmPort database. **(B)** UMAP of the IAG score in all clusters.

We next investigated the immune-associated expression characteristics in KC, and a total of 227 IAGs were screened from 5608 DEGs in KC based on the ImmPort database (**Figure 3A**). The IAG score of each was calculated based on the expression of 227 IAGs using the AUCell package (**Figure 3B**). We found that a high IAG score was mainly found in CSCs and immune cells coloured green-yellow (**Figure 3B**). We also performed GO and KEGG pathway enrichment analyses of DEGs in the CSC and immune cell clusters, respectively (**Figures S1****–** **S2**) and found that DEGs in the CSC cell cluster were mainly enriched in the cell submicrostructure (**Figure S1**). However, the DEGs in the immune cell cluster were mainly involved in immune activation and the immune response (**Figure S2**).

### 3.4 Transcriptional changes and landscape of immune cell infiltration in KC

**Figure 4:**
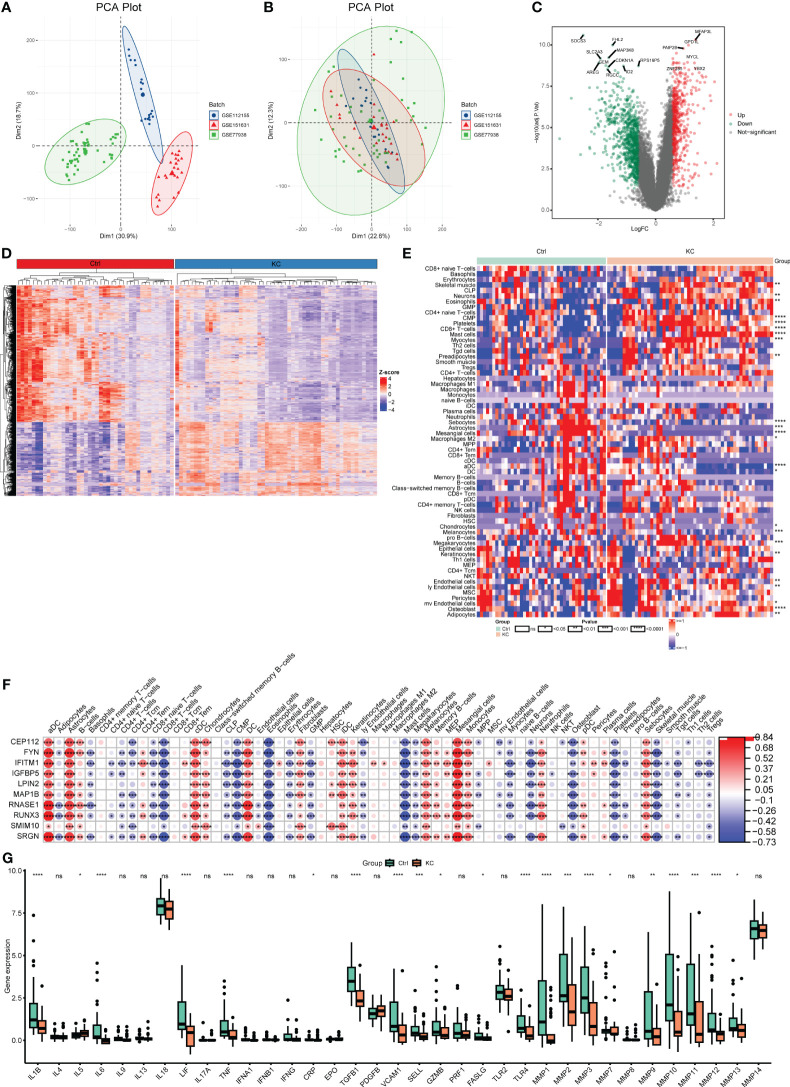
Transcriptional changes and the landscape of immune cell infiltration in KC. **(A, B)** PCA plots of expression profiling in three GEO datasets (GSE151631, GSE112155, and GSE77938) before and after combing. **(C)** Volcano plot of DEGs between KC and health controls based on the bulk RNA-seq data. **(D)** Heatmap of DEGs between KC and health controls based on the bulk RNA-seq data. **(E)** Heatmap of different fractions of immune cells between KC and health controls. **(F)** Heatmap of correlation between immune cells and their marker genes. **(G)** Boxplots of differentially expressed cytokines and MMPs between KC and health controls. *P < 0.05, **P < 0.01, ***P < 0.001, ****P < 0.0001; ns, no significant.

After removing the batch effects, the transcriptomic profiles from three GEO datasets (GSE151631, GSE112155, and GSE77938) were merged and used for subsequent analyses (**Figures 4A, B**). A total of 1362 DEGs (553 upregulated and 809 downregulated) were then identified in KC with the following thresholds: |log2 (FC)| > 0.585 and FDR < 0.05 (**Figures 4C, D**). We further found a marked distinction between different immune cell populations in KC (**Figure 4E**). Skeletal muscle, neurons, eosinophils, CMP, platelets, CD8+ T-cells, mast cells, myocytes, preadipocytes, endothelial cells, ly endothelial cells, megakaryocytes, mv endothelial cells, adipocytes, and osteoblast were significantly increased, but sebocytes, astrocytes, mesangial cells, macrophages M2, aDC, DC, chondrocytes, melanocytes, and keratinocytes were decreased in KC compared with control (**Figure 4F**). Moreover, we explored the different expression levels of cytokines and MMPs, which revealed decreased expression levels of IL-1B, IL-6, LIF, TNF, CRP, TGFB1, VCAM1, SELL, GZMB, FASLG, TLR4, MMP-1, MMP-2, MMP-3, MMP-9, MMP-10, MMP-11, MMP-12, and MMP-13 and upregulated expression levels of IL-5 and MMP-7 in KC patients compared with healthy controls (**Figure 4G**). These data suggested that alterations in the immune system might be involved in KC progression.

### 3.5 Identification of hub IAGs based on WGCNA

**Figure 5:**
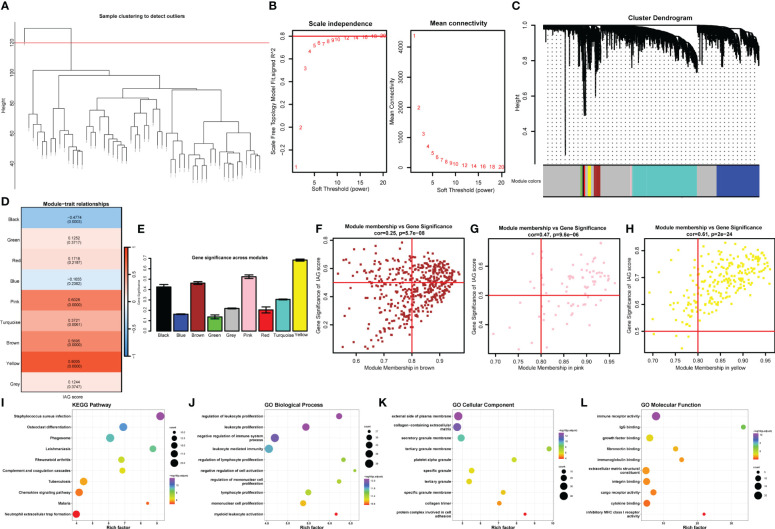
Identification of the hub IAGs based on WGCNA. **(A)** Clustering dendrogram of all samples. **(B)** The scale-free index for various soft-threshold powers (β) and the mean connectivity for various soft-threshold powers. **(C)** Dendrogram of all differentially expressed genes clustered based on the measurement of dissimilarity (1-TOM). The color band shows the results obtained from the automatic single-block analysis. **(D)** Heatmap of the correlation between the module eigengenes and IAG score of KC. **(E)** Histogram of the gene significance across modules. **(F–H)** Scatter plots of correlation of MM with GS in KC. **(I-L)** The bubble plots of KEGG pathways and GO annotation (BP, CC, and MF) of hub genes from significant modules.

To further explore the immune-related features of KC, as shown in **Figure 5A**, all samples from the abovementioned GEO datasets were selected for coexpression analysis. We set the soft threshold to 18 (R ^2^ = 0.8) to construct a scale-free network, and the adjacency matrix and the topological overlap matrix were constructed (**Figure 5B**). All the genes were then distributed into nine modules based on average hierarchical clustering and dynamic tree clipping (**Figure 5C**). Three modules, brown, pink, and yellow, were highly related to the IAG score and were selected as IAG-related modules for further analysis (**Figures 5D, E**). A total of 367 genes with MM > 0.6 and GS > 0.3 were selected as immune-associated hub genes (**Figures 5F, G**). These genes were mainly associated with immune-related pathways, such as neutrophil extracellular trap formation and chemokine signalling pathways (**Figure 5I**), and their GO terms were mainly associated with immune activation and immune response (**Figures 5J–L**).

### 3.6 Selection of diagnostic biomarkers of KC

**Figure 6:**
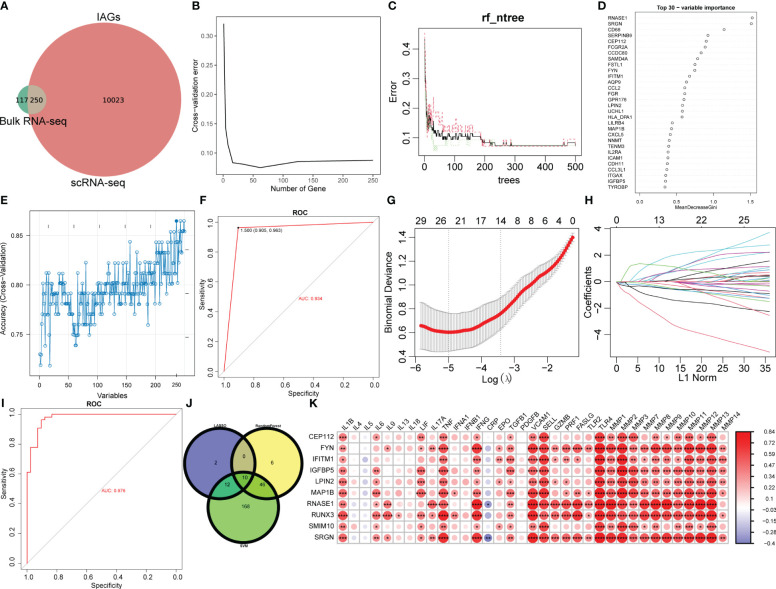
Selection of diagnostic biomarkers for KC. **(A)** Venn plot of differentially expressed IAGs based on WGCNA and DEGs from CSC and immune cell clusters with the highest IAG score. **(B–D)** Characteristic genes were selected using a random forest algorithm. **(E)** Characteristic genes were selected using the SVM algorithm. **(F)** ROC curve has validated the reliability of SVM. **(G, H)** Characteristic genes were selected using LASSO regression analysis. **(I)** ROC curve validated the reliability of the LASSO model. **(J)** Venn plot of diagnostic IAGs in KC by overlapping characteristic genes from three algorithms. **(K)** Heatmap of the correlation of diagnostic IAGs with cytokines and MMPs. *P < 0.05, **P < 0.01, ***P < 0.001, ****P < 0.0001.

To identify immune-related diagnostic biomarkers of KC, the 250 IAGs based on 10273 DEGs with high IAG scores identified from the scRNA-seq data and the 367 hub immune-related genes identified from the bulk RNA-seq data were used for subsequent investigation (**Figure 6A**). Three machine learning algorithms, RF, SVM, and LASSO regression analyses, were utilized to identify 62, 236, and 24 key IAGs, respectively (**Figure 6B-H**). After intersection, 10 IAGs (CEP112, FYN, IFITM1, IGFBP5, LPIN2, MAP1B, RNASE1, RUNX3, SMIM10, and SRGN) were identified in KC (**Figure 6J**). We then investigated the correlation among 10 diagnostic IAGs with 24 cytokines and MMP1-14 and found a strong positive correlation for the 10 IAGs with cytokines and MMP1-14 except IL-4, IL-5, IL-9, IL-13, IL-17A, IL-18, IFNA1, IFNB1, CRP, and PDGFB (**Figure 6K**). These data indicated that the diagnostic genes might regulate immunological features during KC progression.

### 3.7 Development of a nomogram

**Figure 7:**
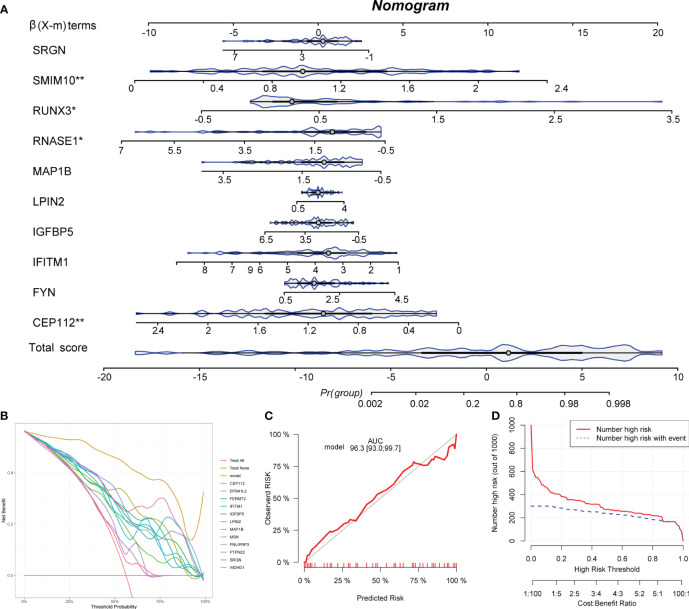
Development of a nomogram. **(A)** A nomogram to predict the KC progression was constructed based on 12 IAGs. **(B)** The decision curve was used to evaluate the accuracy of the nomogram. **(C)** The calibration curve was used to evaluate the sensitivity of the nomogram. **(D)** The clinical impact curve was used to evaluate the clinical usefulness of the nomogram. *P < 0.05, **P < 0.01.

Ten IAGs were incorporated to construct a nomogram to predict KC progression (**Figure 7A**). Each gene corresponded to a score, and the total score from each gene was used to indicate the different risk scores of KC. In the decision curve analysis, the patients diagnosed with KC could clinically benefit from the nomogram (**Figures 7B**). The calibration curve and clinical impact curve revealed the accuracy of the nomogram for predicting KC progression (**Figure 7C, D**).

## 4 Discussion

KC is a progressive corneal disorder that seriously threatens vision, and corneal curvature, corneal topography, corneal thickness, and ocular coherence tomography (OCT) are common parameters used for the clinical diagnosis of visual acuity in KC patients; however, visual acuity is not an ideal predictor of KC symptoms (25, 26). The treatment of KC varies depending on the disease severity and progression. In the early stage, KC can be controlled through contact lenses and corneal collagen cross-linking (27). As the disease progresses, it can only be controlled through corneal transplantation (11). Therefore, exploring sensitive diagnostic markers for the early diagnosis of KC and investigating the regulatory mechanisms are necessary to understand the etiology and pathology of KC.

In the current study, we identified a decreased percentage of the CSC5 cluster and an increased percentage of the CSC6 cluster in KC patients compared with healthy controls, and the CSC and immune cell clusters had higher IAG scores than other cell clusters, suggesting heterogeneity in the function of CSCs and immune cells in KC. Based on the bulk RNA-seq data, we found an obvious distinction between immune cell populations and cytokines as well as MMPs between KC patients and healthy controls. Moreover, we also identified the IAG score-related gene modules and genes in KC. A comprehensive analysis of the scRNA-seq and bulk RNA-seq data identified 250 intersecting IAGs, which were used to select potential diagnostic markers. CEP112, FYN, IFITM1, IGFBP5, LPIN2, MAP1B, RNASE1, RUNX3, SMIM10, and SRGN were ultimately identified and validated as potential diagnostic markers of KC.

ScRNA-seq is a powerful tool that identifies cell heterogeneity and different cell subpopulations based on high-throughput and single-cell level research. This technique has been widely applied in fields such as tumour therapy, reproductive and reproductive health, immunology, neuroscience, and microbiology. Specific markers that can identify eye cell types can aid the discovery of new cell subtypes and the identification of secondary subpopulations that play critical roles in diseases. Constructing cell and gene expression profiles and analysing gene expression and mutations in the eye at the single-cell level allow the exploration of physiological and pathological processes such as eye development and disease progression at high resolution. scRNA-seq has been performed for the analysis of multiple ocular tissues, including the retina (28–30), sclera (31) and limbus (32). Dou et al. obtained central cornea tissues from patients with KC and healthy individuals and elucidated the cell type-specific transcriptional alterations in KC. No significant difference in the composition of various types of cells, including the types of immune cells (23), was found between the two groups. We further analysed the cell subpopulations and found a decrease in CSC5 and an increase in CSC6 in KC. To explore the heterogeneous changes in keratoconus corneas, Dou et al. divided CSCs into different subpopulations and find that different subtypes of CSCs have different biological functions and their susceptibility to keratoconus also varies. Almost all cytokines were enriched in immune cells, and the inflammatory response and extracellular mechanism degradation were increased in Macs/Monos. Multiple interleukins and chemokines were elevated in DCs in KC. Enrichment analysis showed that the DEGs were mainly concentrated in sugar chain synthesis, endoplasmic reticulum protein processing, tight junctions and other cellular submicroscopic structures, suggesting that different types of corneal stromal cells may play different roles in extracellular fibrous tissue reconstruction and that immune cells also play an important regulatory role in this process. However, the samples were all obtained from patients with advanced disease, which limits a comprehensive understanding of Dou’s research. Therefore, we further analysed the three groups of samples from the GEO and found that KC patients exhibited significant differences from the normal control group in terms of cell subsets. KC patients showed significant increases in mast cells, eosinophils, platelets and other cells and decreases in astrocytes, mesangial cells and other cells. Significant differences in DCs, T cells, and other cells were also detected. Bozkurt et al. also found that the platelet/lymphocyte ratio (PLR) was significantly increased in KC and can serve as a biomarker of KC (33). While mast cells and eosinophils generally accumulate in allergic and other immune inflammation, KC is closely related to immune inflammation. Additionally, a significant correlation between characteristic genes and immune cell infiltration was detected and illustrated. We found that the immune-related genes selected in this study were significantly correlated with multiple cell types, mainly positively associated with aDCs, B-cells, astrocytes, cDCs, chondrocytes, DC, iDCs, keratinocytes, melanocytes, monocytes, neutrophils, pDCs, mesangial cells, monocytes, and sebocytes, but negatively associated with adipocytes, basophils, CD4+ T cells, CD4+ Tcm, CLP, CMP, eosinophils, erythrocytes, GMP, mast cells, megakaryocytes, myocytes, neutrophils, osteoblasts, platelets, skeletal muscle, Tgd cells, and Tregs. In the context of diseases associated with keratoconus, mast cells and eosinophils emerge as the most pertinent immune cells related to allergic diseases (34). The extent of immune dysregulation in Down Syndrome (DS) is substantial, encompassing both innate and adaptive immune systems and manifesting anomalies in various immune cells, including T and B cells, monocytes, among others (35). Consequently, we posit that the elevated incidence of keratoconus in patients with allergic diseases and DS may be attributed to the involvement of immunological factors.

In addition, we analysed the expression of cytokines and MMP, IAGs mainly positively associated with IL-1B, IL-6, LIF, TNF, IFNG, TGFB1, VCAM1, SELL, TLR4, and MMP1-14. Surprisingly, we found that the expression of most cytokines and MMP was decreased in KC, which is different from the results of previous studies (36). IL-6 is the most relevant cytokine in the tear fluid of KC, especially in allergic KC. The severity of KC is closely related to the concentration of IL-6 in tear fluid (37). Berger et al. found that the gene expression level of IL-6 mRNA in cultured fibroblasts from KC patients was lower than that of normal individuals, but no difference in protein levels was observed (38). These differences include increased IL-6 levels and decreased IL-12, TNF-α, IFN-γ, IL-4, IL-13 and CCL5 levels in KC compared to control tear fluids. The decreases in IL-12, TNF-α and CCL5 were significant, whereas the IL-13 decrease was significant only in the severe KC group (39). Therefore, differences in the expression of inflammatory factors were found between KC patients and healthy controls. We analysed possible reasons for this divergence, including different patient populations, such as whether they have concomitant atopy and allergies. Second, the severity of KC disease varies, and third, the detection methods vary. However, regardless of the results, the immune system and its stable state are disrupted in KC.

In the present study, we further analysed the immune characteristics in KC and analysed the DEGs obtained from the GEO data analysis after strict threshold setting. The enriched GO biological process showed that the text mining genes(TMGs) were mainly associated with immune receptor activity, cytokine receptor binding, cytokine activity, monocyte differentiation, leukocyte migration and adhesion, whereas the functions of CSCs were mainly enriched in catalytic RNA activity, isomerase activity, collagen binding, myosin binding, and calmodulin binding. The KEGG pathway enrichment analysis revealed that TMGs were associated with the chemokine signalling pathway, NF-KB signalling pathway, and rheumatoid arthritis, among others.

In the present study, 10273 DEGs with high IAG scores identified from scRNA-seq data and 367 hub immune-related genes identified from bulk RNA-seq data were used for subsequent investigation, and 250 IAGs were selected. Then, we used three machine learning algorithms, RF, SVM, and LASSO regression analyses, to identify the key IAGs. Ten hub IAGs, CEP112, FYN, IFITM1, IGFBP5, LPIN2, MAP1B, RNASE1, RUNX3, SMIM10, and SRGN, were ultimately identified in KC. Moreover, a nomogram revealed that SMIM10, RUNX3, RNASE1, and CEP112 significantly contributed to the KC progression.

Centrosomal protein 112 (CEP112) encodes a coiled-coil domain-containing protein that belongs to the cell division control protein 42 effector protein family. In neurons, this protein localizes to the cytoplasm of dendrites and is also enriched in the nucleus, where it interacts with the RNA polymerase III transcriptional repressor Maf1 to regulate gamma-aminobutyric acid A receptor surface expression. In addition, the protein has been identified as a component of the human centrosome. CEP112 can be used as a DNA methylation biomarker associated with the risk of cancer liver metastasis in patients with early-stage CRC. CEP112 malfunction can lead to severe disruption of and increasingly abnormal cell mitosis, leading to malignant transformation (40). Although no literature has reported that CEP112 is related to the progression of KC, based on previous studies and the results of this study, we speculate that CEP112 may acts as a novel marker to participate in the pathologic progression of KC by regulating immune cell infiltration.

FYN, a Src family tyrosine kinase, plays a role in many biological processes, including the regulation of cell growth and survival, cell adhesion, integrin-mediated signalling, cytoskeletal remodelling, cell motility, immune response and axon guidance. FYN is also involved in the processes of corneal neovascularization and corneal epithelial injury repair (41).

Interferon-induced transmembrane protein 1 (IFITM1), the expression of which can be induced by interferon-gamma, encodes a cell surface protein known to influence cell differentiation. IFN-γ affects the homeostasis of the conjunctival epithelium and promotes squamous metaplasia of the conjunctiva. Interferon-gamma can enhance the expression of IFITM1 in the conjunctiva of patients with dry eye syndrome, which may play a role in abnormal terminal differentiation of the epithelium (42).

Insulin-like growth factor-binding protein 5 (IGFBP5), the most conserved member of the IGFBP family in vertebrates, plays a critical role in controlling cell survival, growth, differentiation, and apoptosis. IGBF can enhance the stability of corneal epithelial cells. Studies have shown that IGFBP5 expression is decreased 14-fold in cultured KC stromal cells (43).

LPIN2 is a member of the lipin family of enzymes which act the key effectors in the biosynthesis of lipids. LPIN2 mutations link to increased IL-1β and activated NLRP3 inflammasome and may be related to myopia (44).

Microtubule-associated protein 1B (MAP1B) facilitates the tyrosination of alpha-tubulin in neuronal microtubules. Phosphorylated MAP1B may play a role in the cytoskeletal changes that accompany neurite extension. It is possible that MAP1B binds to at least two tubulin subunits in the polymer, and this bridging of subunits might be involved in nucleating microtubule polymerization and in stabilizing microtubules (45). Although there are few studies on MAP1B, and even almost no reports in KC, we still tracked the expression trace of MAP1B in corneal epithelial cells, and our study filled the gap in the study of corneal epithelial cells in KC.

Ribonuclease A family member 1 (RNASE1) is one of the best-characterized vertebrate-specific proteins that can regulate intracellular or extracellular RNA metabolism, antiviral, antibacterial, and antifungal activities, neurotoxicity, promotion of cell proliferation, anti-apoptosis, and immune regulatory abilities. RNASEs have been found to be involved in the pathogenesis of many diseases, such as infection, immune dysfunction, neurodegeneration, cancer, and cardiovascular disease (46).

The runt box (RUNX) is a highly conserved DNA-binding and protein-protein interaction domain that defines a family of heterodimeric transcription factors with essential roles in metazoan development. RUNX3 controls neurogenesis in the dorsal root ganglia and cell proliferation in the gastric epithelium and is frequently deleted or silenced in human gastric cancer (47).

Small integral membrane 10 (SMIM10) is a gene located on the X chromosome that encodes an 83-aa protein of unknown function. In melanoma cells, this gene encodes a mitochondrial protein that selectively downregulates BRAFV600E RNA and protein levels by acting indirectly at the posttranscriptional level (48).

Serglycin (SRGN) is a hematopoietic cell granule proteoglycan that functions in the formation of mast cell secretory granules, mediates the storage of various compounds in secretory vesicles, and thus plays a role in cell apoptosis mediated by cytotoxic cell particles. SRGN might serve as a promising biomarker with high specificity and sensitivity in diabetic retinopathy diagnosis and progression (49).

The genes previously associated with the onset of keratoconus include corneal thickness-related genes such as ADAMSTS6 and ARID5B (50) and cell adhesion-related genes such as LAMB3, LAMA3 and LAMA1 (51). Research on immune related genes in keratoconus is limited. Chen et al. investigated the immune-related pattern differences of keratoconus, including gene expression, signaling pathways, and immune cell infiltration. Ultimately, it was found that the chemokine receptors CCR2 and CCR5, as well as F2RL1 and CXCL5, may be involved in the immune regulation process of keratoconus (52). None of these genes in our study have been reported to be directly associated with KC, which to some extent indicates the gap in KC-related immunology research. Some of these genes, such as RNASE, IFITM1, SMIM10, and SRGN, are enriched in immune cells. These genes and their encoded proteins are generally involved in processes related to cell stability, including cell metabolism and damage repair. The current research on these genes mainly focuses on corneal epithelial cells. Damage to corneal epithelial cells leads to cytokine release, differentiation of myofibroblasts, changes in corneal shape, corneal biomechanics, and thinning of corneal tissue (53). In KC, abnormal differentiation signals are observed in corneal superficial cells. A recent study observed a reduced pool of limbal suprabasal cells and found that signalling pathways that affect metabolic changes and cell contact in epithelial and stromal cells are affected in KC (54). Further research is needed to determine whether these genes and their encoded proteins have an impact on other corneal cells.

In this study, we analysed the correlation between the ten IAGs and cytokines, as well as MMPs. We found a significant relationship, especially with MMPs. MMPs have the ability to degrade collagen and other extracellular matrix (ECM) proteins, which are essential components of the cornea. Over the past two decades, an increasing number of publications have indicated a link between MMPs and keratoconus. Corneal MMPs, especially MMP-1 and MMP-9, are upregulated in the corneal tissues and tears of KC patients. There is also evidence suggesting a causal relationship between inflammation and the increased production of MMPs observed in KC. This inflammation might be caused by mechanical trauma from wearing contact lenses or rubbing the eyes (55). This also suggests that the IAGs we identified are persuasive.

Although our findings demonstrate that CSCs and immune cells play a key role in KC and that immunological processes might be involved in KC progression and identify potential diagnostic markers of KC, these results have not been validated using experimental methods. In future studies, we will collect tear solutions from patients with KC and inflammatory eye disease as well as healthy controls and will then detect the expression levels of these markers. Moreover, the tear solution tested an alternate concept for safely and conveniently propelling a future diagnosis and its crew away from the problem of collecting corneal tissue.

In conclusion, we identified differences in different corneal stromal cell subtypes between KC patients and healthy controls by combining single-cell and bulk transcriptional data, and the CSC and immune cell clusters had higher IAG scores than other cell clusters. We found an obvious distinction between immune cell populations and cytokines as well as MMPs between KC patients and healthy controls. By applying a series of bioinformatics approaches, 10 IAGs (CEP112, FYN, IFITM1, IGFBP5, LPIN2, MAP1B, RNASE1, RUNX3, SMIM10, and SRGN) were identified as potential prognostic genes for KC that were significantly associated with the expression levels of cytokines and MMP1-14. These genes are mostly related to cell stability, and some are enriched in immune cells. Our study confirms the role of immune-related factors in the development of KC and elucidates the pathogenic mechanism of cell stability in KC from another perspective. The absence of experimental validation is a limitation of this study, and further studies are needed.

## Data availability statement

The original contributions presented in the study are included in the article/**Supplementary Material**. Further inquiries can be directed to the corresponding author.

## Author contributions

XN and YY: conceived and designed the study. XN: Writing- Original draft preparation, Methodology, Software. MX: Methodology, Data curation, Investigation. JZ and SZ: Methodology, Data curation. All authors contributed to the article and approved the submitted version.
