# Supplementary figures and images for: Identification of the immune-associated characteristics and predictive biomarkers of keratoconus based on single-cell RNA-sequencing and bulk RNA-sequencing
Source: Front Immunol. 2023 Oct 27;14:1220646. doi: 10.3389/fimmu.2023.1220646 (PMC10641680; doi:10.3389/fimmu.2023.1220646)

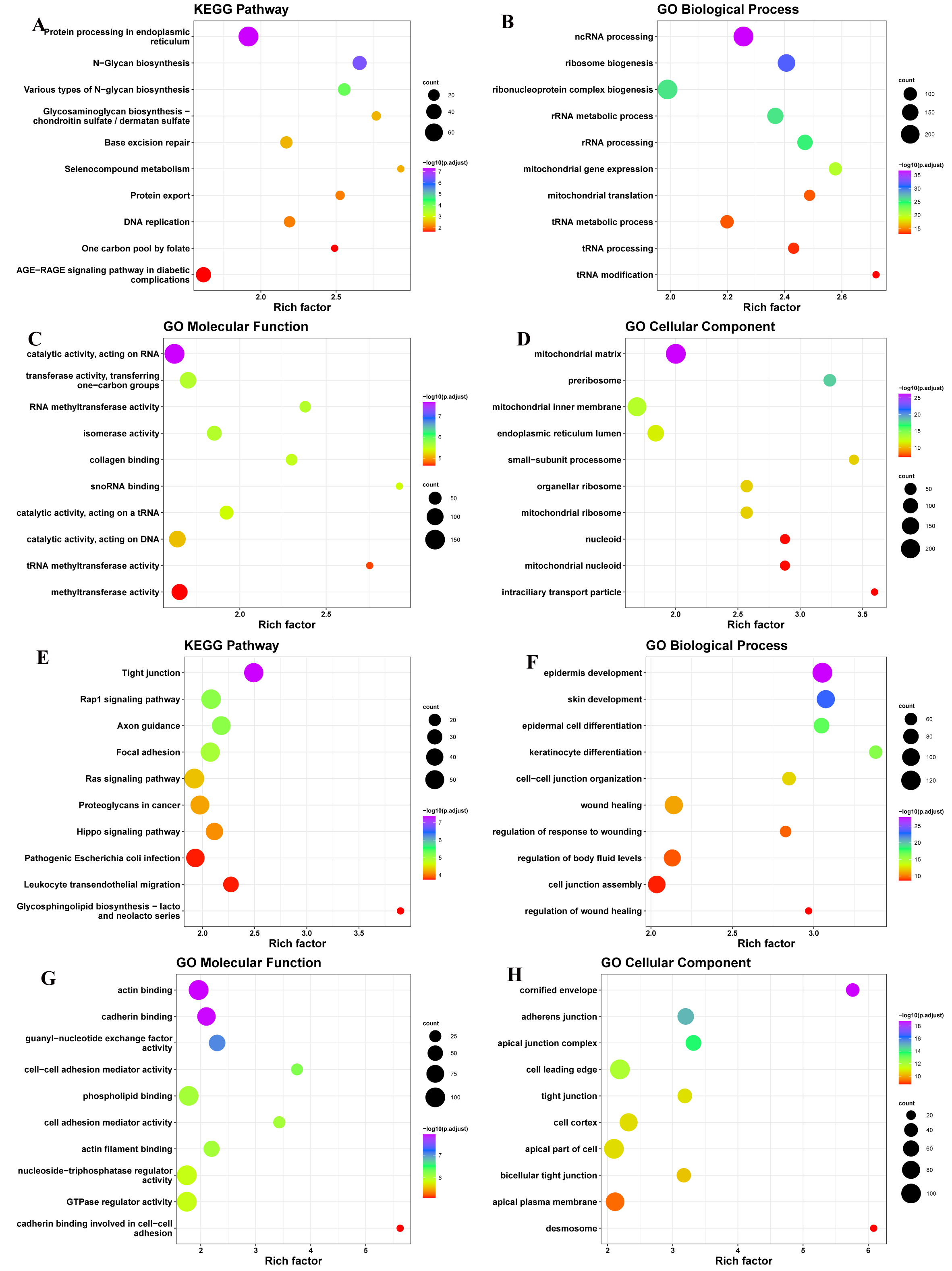

Supplement: Supplementary file 8 [file Image_1.tif]

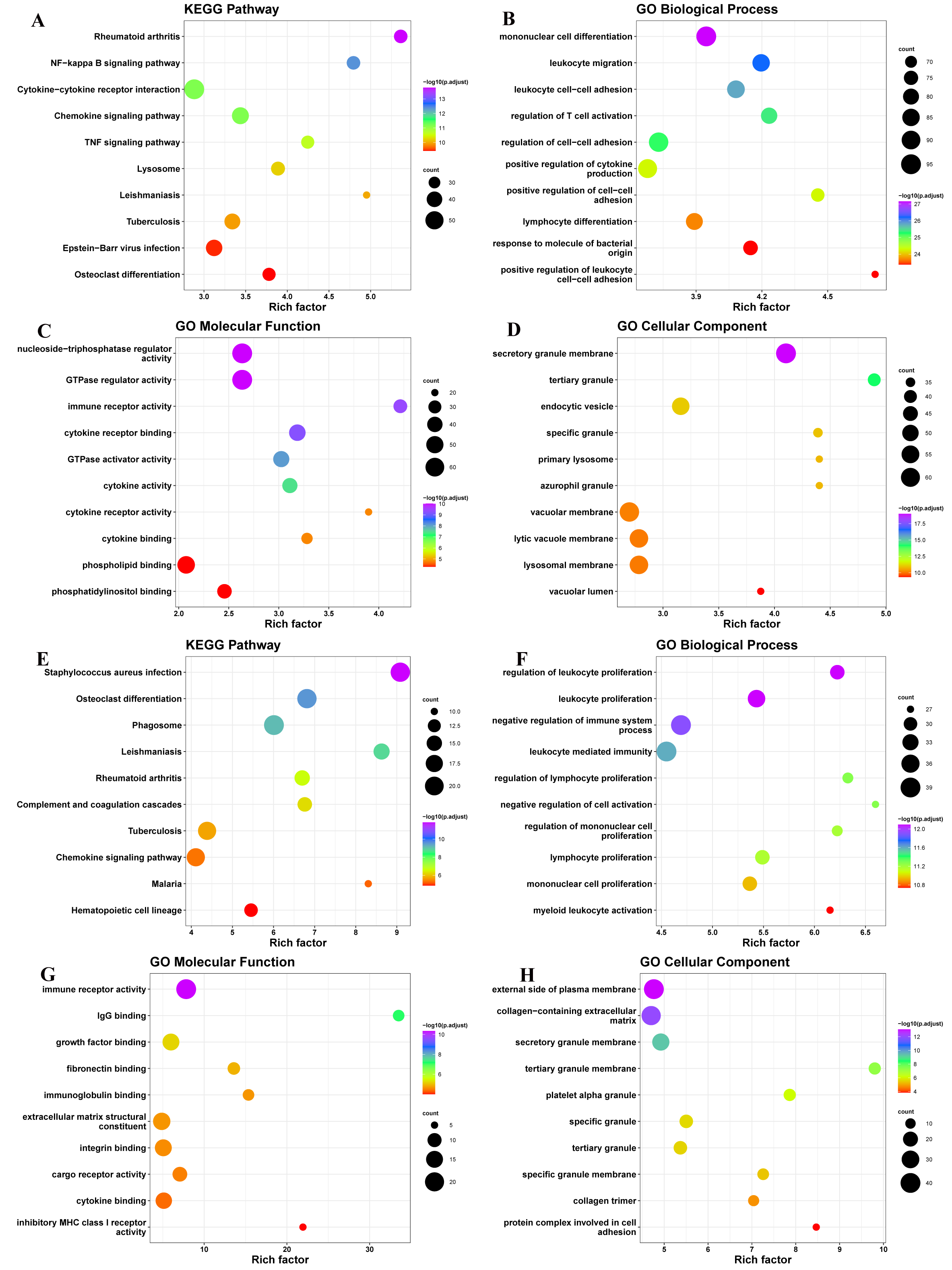

Supplement: Supplementary file 9 [file Image_2.tif]
